# Supplementary material for: Predicting visual function by interpreting a neuronal wiring diagram
Source: Nature. 2024 Oct 2;634(8032):113–23. doi: 10.1038/s41586-024-07953-5 (PMC11446822; doi:10.1038/s41586-024-07953-5)
Supplement: Supplementary file 6 — CRF and ERF predictions for individual TmY4 and TmY9 cells. Analogous to Supplementary Data 3, but for TmY target types. Shown are the top four monosynaptic pathways, the strongest pathway passing through each of the top ten intermediary types (ranking from Extended Data Fig. 7), and the trisynaptic pathway Tm1–TmY–Dm3–TmY (see the section entitled Prediction of spatial normalization). [file 41586_2024_7953_MOESM6_ESM.zip › DataS4/TmY9qΓèÑ/720575940617744182.pdf]

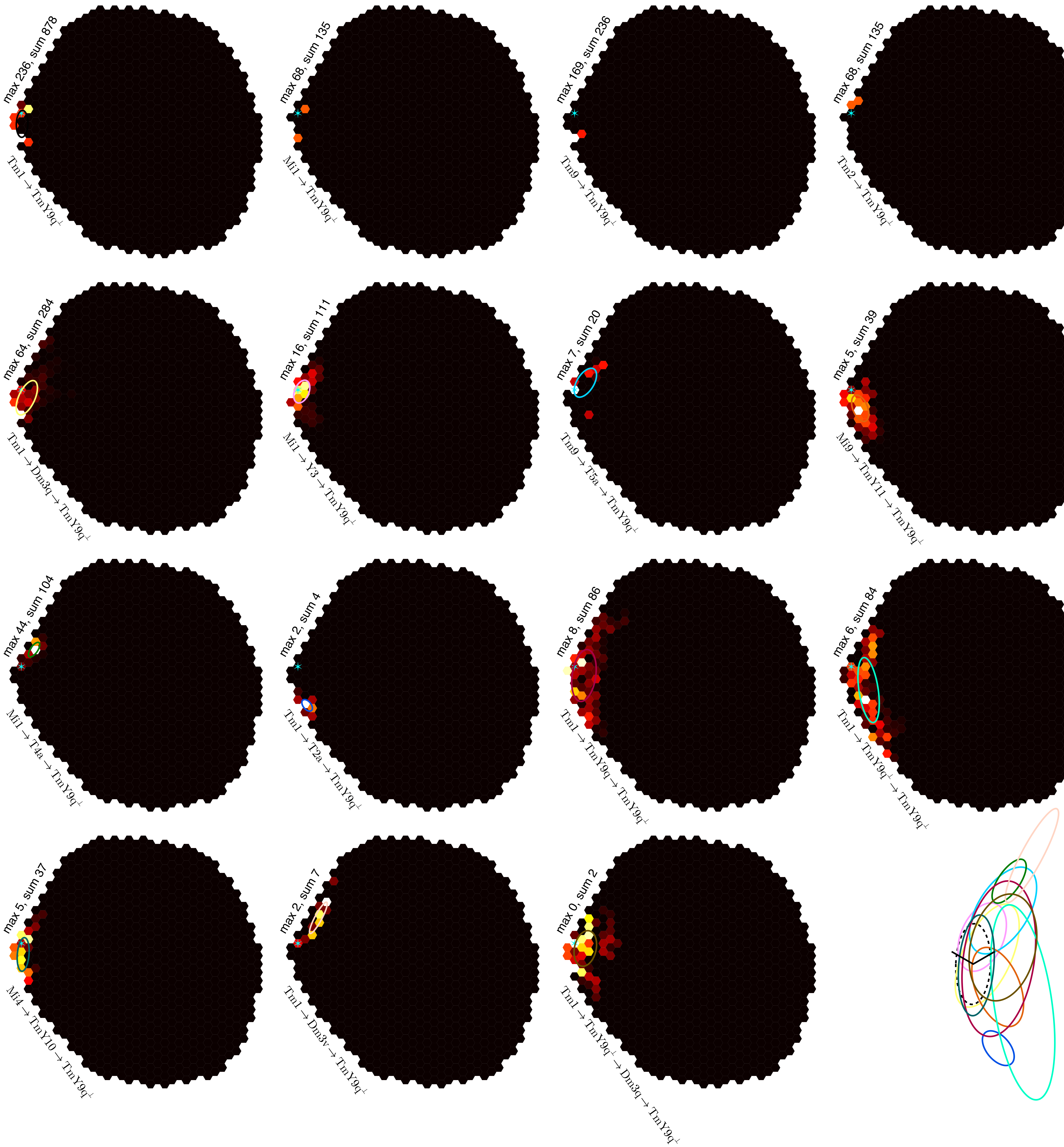
max 236, sum 878
Tm1 → TmY9q⊥
max 68, sum 135
Mi1 → TmY9q⊥
max 169, sum 236
Tm9 → TmY9q⊥
max 68, sum 135
Tm2 → TmY9q⊥
max 64, sum 284
Tm1 → Dm3q → TmY9q⊥
max 16, sum 111
Mi1 → Y3 → TmY9q⊥
max 7, sum 20
Tm9 → T5a → TmY9q⊥
max 5, sum 39
Mi9 → TmY11 → TmY9q⊥
max 44, sum 104
Mi1 → T4a → TmY9q⊥
max 2, sum 4
Tm1 → T2a → TmY9q⊥
max 8, sum 86
Tm1 → TmY9q → TmY9q⊥
max 6, sum 84
Tm1 → TmY9q⊥ → TmY9q⊥
max 5, sum 37
Mi4 → TmY10 → TmY9q⊥
max 2, sum 7
Tm1 → Dm3v → TmY9q⊥
max 0, sum 2
Tm1 → TmY9q⊥ → Dm3q → TmY9q⊥
